# Three-year changes in sex judgment using color fundus parameters in elementary school students

**DOI:** 10.1371/journal.pone.0295123

**Published:** 2023-11-30

**Authors:** Takehiro Yamashita, Ryo Asaoka, Hiroto Terasaki, Naoya Yoshihara, Naoko Kakiuchi, Taiji Sakamoto

**Affiliations:** 1 Department of Ophthalmology, Kagoshima University Graduate School of Medical and Dental Sciences, Kagoshima-shi, Kagoshima, Japan; 2 Department of Ophthalmology, Seirei Hamamatsu General Hospital, Hamamatsu, Shizuoka, Japan; 3 School of Nursing, Seirei Christopher University, Hamamatsu, Shizuoka, Japan; 4 Nanovision Research Division, Research Institute of Electronics, Shizuoka University, Hamamatsu, Shizuoka, Japan; 5 The Graduate School for the Creation of New Photonics Industries, Hamamatsu, Shizuoka, Japan; Federal University of Rio de Janeiro: Universidade Federal do Rio de Janeiro, BRAZIL

## Abstract

**Purpose:**

In a previous cross-sectional study, we reported that the sexes can be distinguished using known factors obtained from color fundus photography (CFP). However, it is not clear how sex differences in fundus parameters appear across the human lifespan. Therefore, we conducted a cohort study to investigate sex determination based on fundus parameters in elementary school students.

**Methods:**

This prospective observational longitudinal study investigated 109 right eyes of elementary school students over 4 years (age, 8.5 to 11.5 years). From each CFP, the tessellation fundus index was calculated as red/red + green + blue (R/[R+G+B]) using the mean value of red-green-blue intensity in eight locations around the optic disc and macular region. Optic disc area, ovality ratio, papillomacular angle, and retinal vessel angles and distances were quantified according to the data in our previous report. Using 54 fundus parameters, sex was predicted by L2 regularized binomial logistic regression for each grade.

**Results:**

The right eyes of 53 boys and 56 girls were analyzed. The discrimination accuracy rate significantly increased with age: 56.3% at 8.5 years, 46.1% at 9.5 years, 65.5% at 10.5 years and 73.1% at 11.5 years.

**Conclusions:**

The accuracy of sex discrimination by fundus photography improved during a 3-year cohort study of elementary school students.

## Introduction

Artificial intelligence (AI), especially deep learning approaches [1], has enabled the automated identification of ocular diseases such as diabetic retinopathy [2–8] and glaucoma [2, 9–16], using only fundus photographs. Notably, deep learning algorithms can identify the sex of an individual using fundus photographs, with an area under the receiver operating characteristic curve (AUROC) of 0.93 (dataset: UK biobank) [17] or 0.97 (dataset: UK Biobank and EyePACS) [18]. These results suggest that there are several factors with sex differences in the fundus, such as the human face [19]. However, there are few studies about sex differences in fundus photographs.

Previously, we found that a combination of known clinical parameters, such as the angle or trajectory of retinal vessels [20–25], location and shape of the optic disc [26, 27], and color intensity in the peripapillary area [28–30] in color fundus photographs (CFPs) are useful for identifying the sex of individuals. Using these fundus parameters, the AUROC value obtained using ridge binomial logistic regression with leave-one-out cross validation [31, 32] was 77.9% for young adults [33]. This method also allows us to identify specific sex differences in fundus parameters. It was found that the female fundus had higher green and blue intensities and a more oval-shaped disc than the male fundus, and the supratemporal (ST) retinal artery (RA) was closer to the fovea in females.

We also reported a sex discrimination accuracy in children (8.5 years old), where the AUROC was 63.2% when same ridge binomial logistic regression approach was used [34]. It was found that the fundus of female children had a more oval-shaped disc and higher green and blue intensities on the nasal side of optic disc than the fundus of male children. Although it is widely acknowledged that the fundus appearance changes with increasing age [23], it is unclear how and when sex differences appear in the fundus parameters. Therefore, we conducted a longitudinal study to investigate the sex discrimination accuracy before and during puberty by examining the fundus parameters of children from the age of 8 for 4 years [35, 36].

## Methods

### Ethics statement

All procedures used in this study conformed to the tenets of the Declaration of Helsinki and were approved by the Ethics Committee of Kagoshima University Hospital. Written informed assent and informed consent were obtained from all the participants and their parents. This study was registered with the University Hospital Medical Network clinical trials registry (No. UMIN000015239).

### Participants and measurements

This study was part of a longitudinal, prospective, cross-sectional, observational study of third grade students who were 8–9 years old at the first examination [23, 34]. The students attended the Elementary School of the Faculty of Education of Kagoshima University. There were 144 students in the third grade. Informed consent was obtained from 122 (87.4%) students and their parents. The students were examined from November 17 to December 18, 2014 in the initial year and were examined during the same period annually for 4 years until December 4, 2017. Six students were excluded due to truancy or transfer. Seven eyes were excluded because of the difficulty in measuring the fundus parameters. In the end, the right eyes of 109 individuals were used for the analyses. Color fundus photographs were taken with the 3D OCT-1 Maestro (Topcon, Tokyo, Japan). Data were accessed for research purposes from December 4, 2017 to March 31, 2023.

#### Measurement of fundus parameters

**Fig 1:**
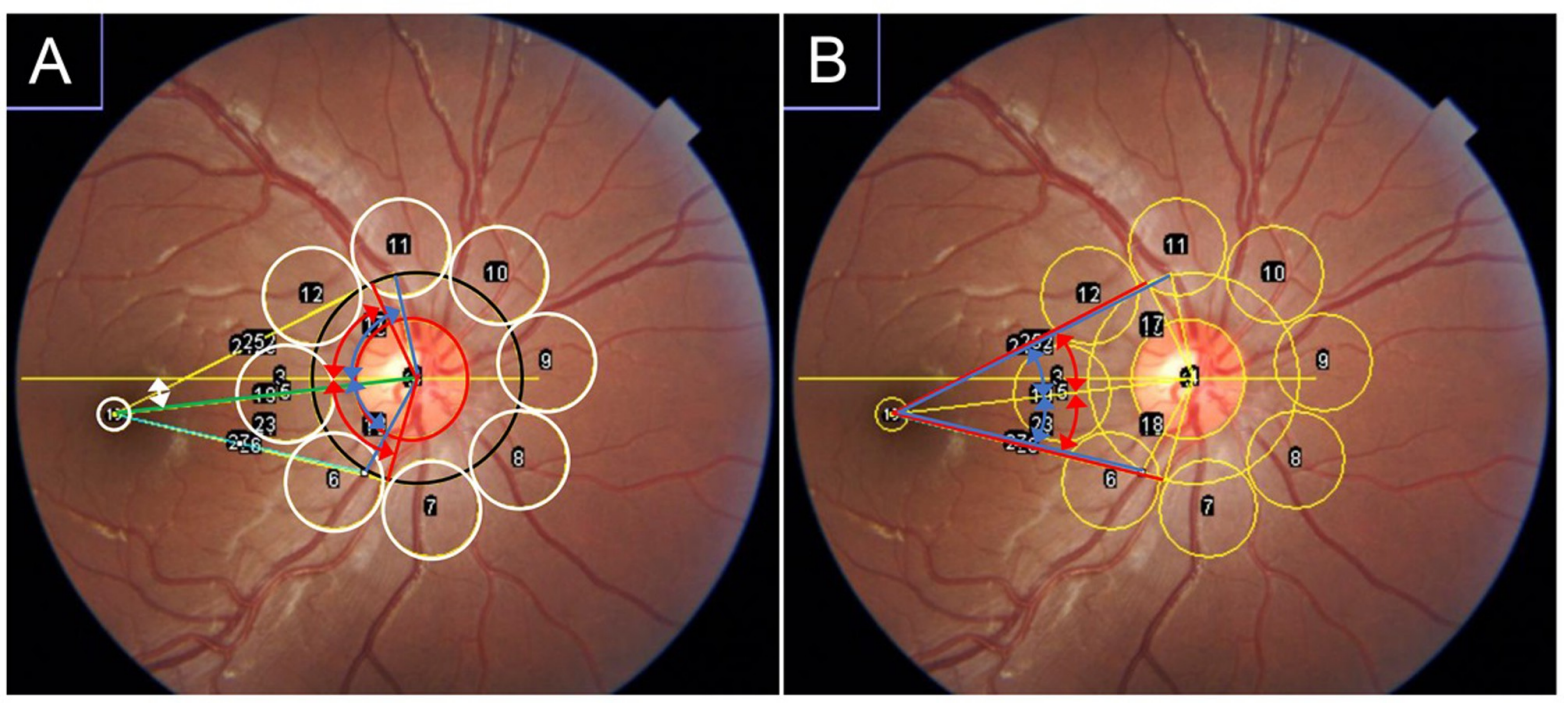
Measurement of fundus parameters. A total of 54 fundus parameters related to location of retinal vessel and color tone of the retina were measured. A) Red-green-blue intensity and the tessellation fundus index (TFI) were calculated in each of the eight locations around the optic disc and fovea (white circles). Optic disc area and ovality ratio were measured using the red circle. The white double arrow indicates the papillomacular angle (PMA). The green line shows the papillomacular distance (PMD). The red double arrows indicate the supratemporal, infratemporal, and total retinal artery angles (ST-RA, IT-RA, T-RA). The blue double arrows indicate the supratemporal, infratemporal, and total retinal vein angles (ST-RV, IT-RV, T-RV). B) The red lines show the supra- and infratemporal artery-fovea distance (STAFD, ITAFD). The blue lines indicate the supra- and infratemporal vein-fovea distance (STVFD, ITVFD). The red double arrows show the supra- and infratemporal artery-fovea angle (STAFA, ITAFA). The blue double arrows indicate the supra- and infratemporal vein-fovea angle (STVFA, ITVFA).

A total of 54 fundus parameters were measured, as in our previous research (Fig 1) [34]. Using the CFP images, a 520-pixel circle was centered on the optic disc center, and the intersections of this circle and the ST or infratemporal (IT) major RAs were identified. Then, the angles between the ST and IT RAs and the temporal horizontal line were measured (T-RA, ST-RA and IT-RA, respectively). Similarly, the angle between the ST or IT major retinal vein (RV) and the temporal horizontal line were measured (T-RV, ST-RV and IT-RV, respectively) [20, 21]. The distances between the intersections and the fovea, supra temporal artery-fovea distance (STAFD), supra temporal vein-fovea distance (STVFD), infra temporal artery-fovea distance (ITAFD), and infra temporal vein-fovea distance (ITVFD) were also quantified. Then, the angles between these lines and the fovea-optic disc line were measured (supra temporal artery-fovea angle (STAFA), supra temporal vein-fovea angle (STVFA), infra temporal artery-fovea angle (ITAFA), infra temporal vein-fovea angle (ITVFA)). The papillomacular distance (PMD), i.e., the distance between the optic disc center and the fovea [37], and the papillomacular angle (PMA), i.e., the angle formed by the papillomacular distance line and horizontal line, were recorded [26]. The optic disc area was quantified by number of pixels in optic disc. The degree of ovality (ovality ratio) was determined by dividing the minimum disc diameter by the maximum disc diameters [27].

The mean values of red, green, and blue intensities within each area were calculated as follows: First, eight 240-pixel circles were set around the optic disc, where the center of the lateral circle was placed on the line between the macular and the center of the optic nerve head. One 80-pixel circle was set at the fovea. The tessellation fundus index (TFI) was calculated using the mean red intensity (R), mean green intensity (G), and mean blue intensity (B) in each of the eight locations as follows: TFI = R/(R + G + B) [28–30].

All these measurements were conducted using CFP images and the ImageJ software (ImageJ version 1.47, National Institutes of Health, Bethesda, MD; available at: http://imagej.nih.gov/ij/). The macro function of ImageJ enabled semiautomated calculation of the above-described CFP parameters; all the 54 parameters were automatically calculated when the locations of the fovea, center of the optic nerve head, and crossing points of supralateral or inferolateral RA or RV were decided [34].

### Statistical analyses

The sex difference in each fundus parameter was evaluated for each grade by the Mann–Whitney U test. As these were exploratory investigations, adjustment for multiple comparisons was not applied [38]. Next, the optimal model for sex was determined using binomial logistic regression with regularization with the 54 parameters above described. Ordinal statistical models, such as linear or binomial logistic regression, may be over-fitted to the original sample, especially when the number of predictor variables is large, such as in the current study (54 parameters of 109 samples). The application of a shrinkage method so that the sum of the absolute values of the regression coefficients is regularized can mitigate these problems in linear/logistic modeling [39, 40]. This method has been used in several different fields, ranging from the analysis of human perception to genetic analysis [41, 42], including our previous studies that have demonstrated the usefulness of this approach for patients with glaucoma [43, 44]. Specifically, in the case of binomial logistic regression with L2 regularization (Ridge binomial logistic regression), the penalized version of the log-likelihood function to be maximized is estimated using the following formula:

$$\sum_{i=1}^{n}\left[\left(yixi\beta -\log\left(1+e^{xi\beta }\right)\right]-\lambda \sum_{j=1}^{p}\beta_{j}^{2}\right.$$

where *xi* is the *i*-th row of a matrix of *n* observations, with *p* predictors, β is the columns vector of the regression coefficients, and λ represents the penalty applied. This formula becomes identical to the ordinary binomial logistic regression when λ = 0. The yielded optimal model enables direct observation of the effect of selected parameters, unlike deep learning.

Next, we evaluated the diagnostic performance of the ridge binomial logistic regression approach using the leave-one-out cross-validation method [41], in which a single observation from the original sample was used as the validation data, and the remaining observations (108 participants) were used as training data. This procedure was repeated 109 times such that each sample was used once as the validation data. The diagnostic accuracy was evaluated using the AUROC. All statistical analyses were performed using SPSS Statistics 19 for Windows (SPSS Inc., IBM, Somers, New York, USA) and the statistical programming language R (ver. 3.1.3, The R Foundation for Statistical Computing, Vienna, Austria).

## Results

**Fig 2:**
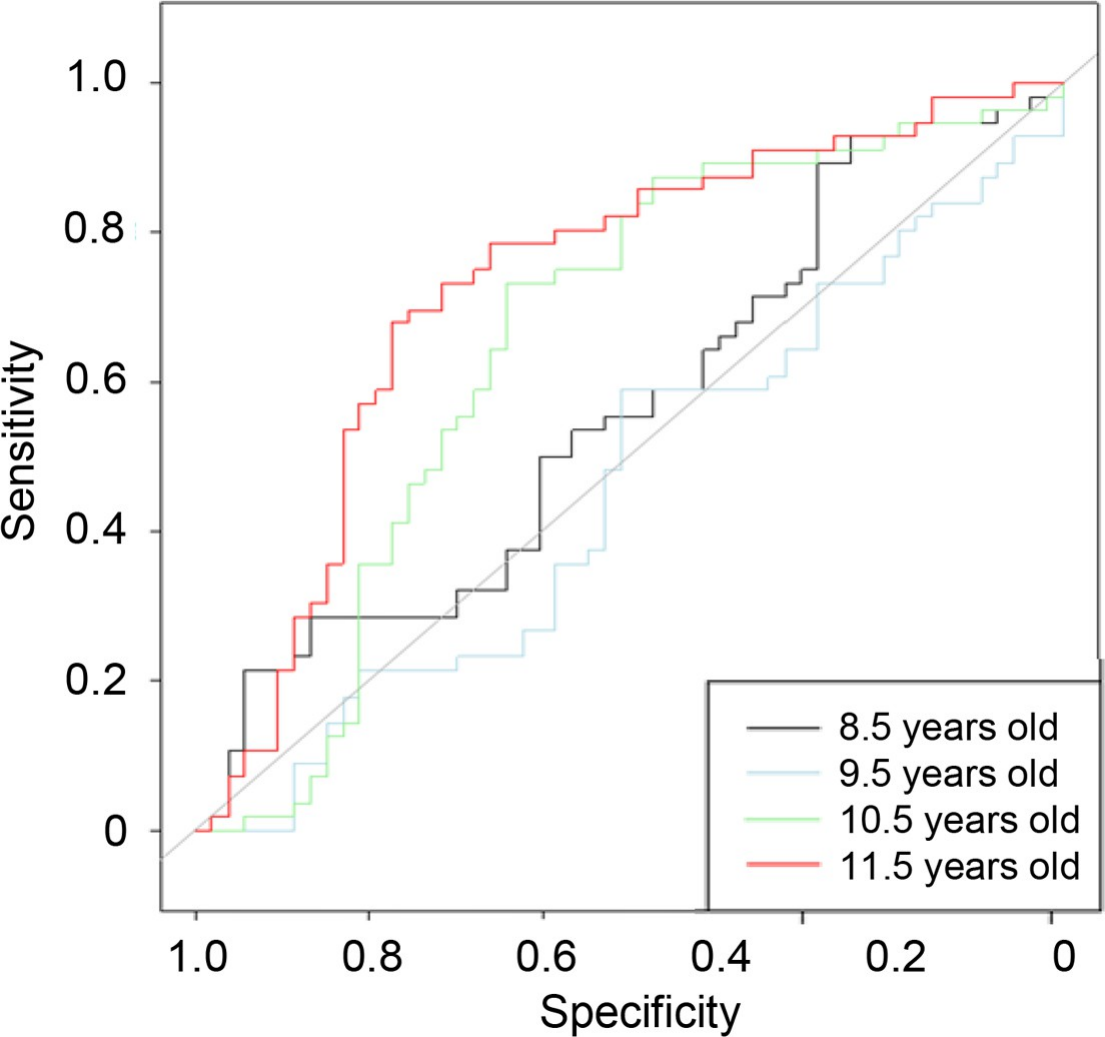
The receiver-operating characteristic curve obtained using ridge binomial logistic regression. The area under the receiver-operating characteristic curve (AUROC) was 56.3% for 8.5 years (black line), 46.1% for 9.5 years (blue line), 65.5% for 10.5 years (green line), and 73.1% for 11.5 years (red line).

The right eyes of 53 boys and 56 girls were analyzed. The mean ± standard deviation and p value of sex differences (Mann–Whitney U test) for each fundus parameter are shown in **S1 Table**. All raw data was available in S2 Table. The AUROC for sex discrimination was 56.3% for the age of 8.5 years, 46.1% for 9.5 years, 65.5% for 10.5 years, and 73.1% for 11.5 years (Fig 2). The AUROC value significantly increased from 9.5 to 10.5 years (p = 0.010) and from 10.5 to11.5 years (p = 0.002; Delong’s method with Holm’s method for the adjustment for multiple comparisons).

**Fig 3:**
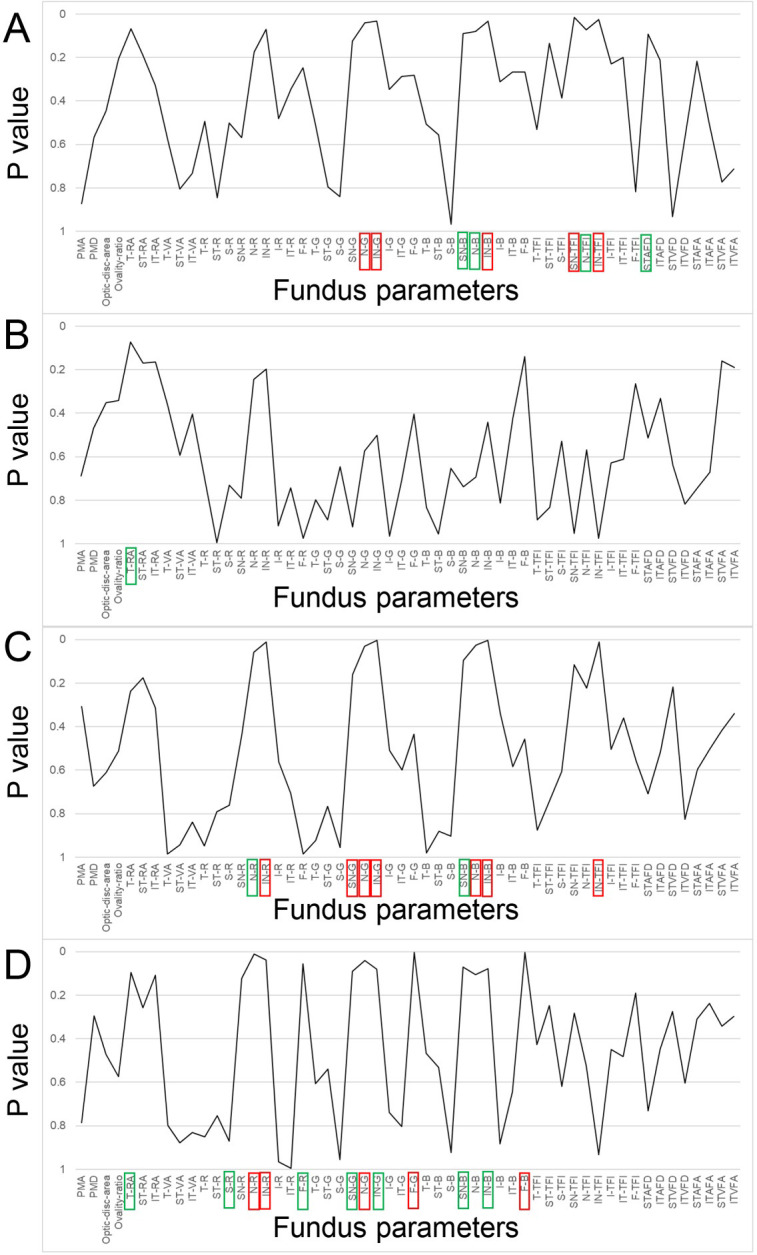
P values from Mann–Whitney U test for sex differences in fundus parameters for each grade (A: 8.5, B: 9.5, C: 10.5, D: 11.5 years old). The red square means a p-value of <0.05, green squares mean a p-value <0.1. PMA: papillomacular angle, PMD: papillomacular distance, T-RA: total retinal artery angle, ST-RA: supratemporal retinal artery angle, IT-RA: infratemporal retinal artery angle, T-VA: total vein angle, ST-VA: supratemporal vein angle, IT-VA: infratemporal vein angle, T: temporal, ST: supratemporal, S: superior, SN: supranasal, N: nasal, IN: Infranasal, I: inferior, IT: infratemporal, F: foveal, R: red intensity, G: green intensity, B: blue intensity, TFI: tessellation fundus index, STAFD: supratemporal artery fovea distance, ITAFD: infratemporal artery fovea distance, STVFD: supratemporal vein fovea distance, ITVFD: infratemporal vein fovea distance, STAFA: supratemporal artery fovea angle, ITAFA: infratemporal artery fovea angle, STVFA: supratemporal vein fovea angle, ITVFA: infratemporal vein fovea angle.

Fig 3 shows the p values from the Mann–Whitney U test for sex differences in fundus parameters for each grade. The sex differences in artery angle, peripapillary nasal and foveal colors, and TFI gradually increased, and the sex differences in temporal-to-inferior peripapillary color gradually decreased.

**Fig 4:**
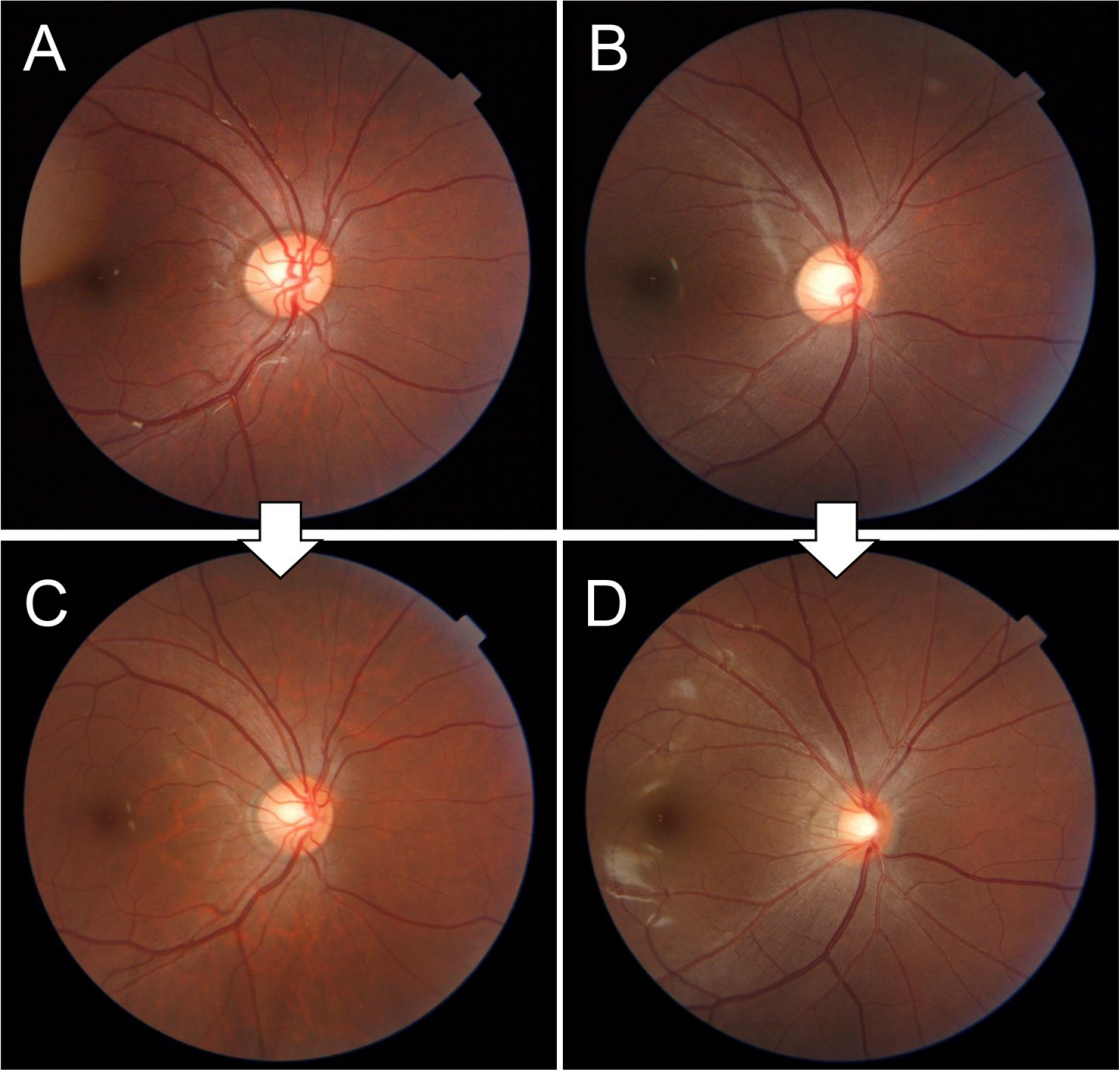
Representative fundus photographs of a boy at age 9 (A) and 12 (C) years old and of a girl at age 9 (B) and 12 (D) years old. The fundus of the girl (**D**) is more bluish and greenish than that of the boy (**C**). The fundus of the boy (**C**) is more tessellated than that of girl (**D**).

**Fig 4** shows the typical eyes of a boy at age 9 (**A**) and 12 (**C**) years old and of a girl at age 9 (**B**) and 12 (**D**) years old. The fundus of the girl (**D**) has more bluish and greenish colors than that of the boy (**C**). The fundus of the boy (**C**) is more tessellated than that of the girl (**D**).

## Discussion

In this study, the sex determination rate based on fundus parameters was found to increase from 56.3% at age 8.5 years to 73.1% at age 11.5 years. Since children start developing secondary sexual characteristics around age 10 [35, 36], the sex differences in fundus photographs can be attributed to the secondary sexual characteristics. The fundus parameters that produced sex differences were similar to those previously reported in young adults in their 20s [33]: girls tended to have more bluish-green hues in the fundus than boys and the retinal arteries were closer to the fovea in girls, whereas boys tended to have a more tessellated fundus than girls (Figs 3 and **4**). As age increases, sex difference in the foveal color appears (Fig 3). The fovea is the focus in the attention heat map analysis of deep learning AI sex determination [18], and sex determination using the Kumejima epidemiological study was also consistent with a stronger foveal green color in females compared to males. In addition to the peripapillary coloration [45], a gradual sex difference in the foveal color is a characteristic of this period. Regarding young adults, females had a more oval-shaped optic disc than males [33]; however, these sex differences were not evident in the present study. Sex differences in the optic disc shape appear after the age of 12 years.

There were only five parameters that were significantly different between males and females, even at 11.5 years old, despite the relatively high AUROC (73.1%) for sex discrimination. Furthermore, even for parameters with significant differences, the distribution of boys overlapped with that of girls, indicating that the sex determination ability was limited when based on a single parameter. Similar results were observed for the 112 young adult eyes, with a judgment rate of 77.9% [33], and the 119 eyes of 8.5-year-olds, with a judgment rate of 63.1% [34]. The sex judgment rate increased by including not only the fundus parameters with significant differences but also those without a significant difference. The fact that deep learning AI guessed the sex from fundus photographs alone with a probability as high as 97% [18] was a surprise to ophthalmologists because they had only seen fundal differences according to sex based on a small number of parameters and had not realized that it was possible to determine sex from fundus photographs. On the other hand, AI does not have the preconceptions that humans have; hence, it can be said that AI can determine a person’s sex from fundus photographs.

Our results show that even if each parameter has a small significant difference, a comprehensive judgment can be made using a large number of parameters collected from a single image. In fact, deep learning AI can estimate not only the sex but also age, blood pressure [18], refraction [46], and axial length [47] from fundus photographs alone with small errors. These results cannot be explained by one or a few fundus features, and high accuracy may be achieved by comprehensive judgment using a large number of fundus parameters. Attention heat maps allow AI to highlight areas of particular interest in an image, but even if a user quantifies only those areas, the distribution overlaps and accuracy of judgment will be low owing to the small number of parameters [48, 49]. Therefore, to understand how AI estimates sex, age, blood pressure, refraction, and ocular axis length from fundus photographs, it is beneficial to recognize and quantify as many features as possible by humans and perform comprehensive regression analysis, as carried out in this study.

In the present study, the sex determination rate decreased from 56.3% to 46.1% at 8.5 to 9.5 years of age, and then gradually increased with increasing age. This result suggests that sex cannot be determined from these fundus parameters until the age of 9.5 years. Because there are few fundus parameters with large sex differences (Fig 3). Another possibility, considering that around 10 years of age is when children develop secondary sexual characteristics such as enlargement of breasts in females and enlargement of the larynx with deepening of voice in males [35, 36], one hypothesis can be raised. The sex differences between boys and girls below 10 years of age is rather different from the sex differences between adult men and women. It is possible that the lack of sexual development up to the age of 8.5 years is offset by the changes due to secondary sexual characteristics around the age of 10 years, resulting in a temporary drop in the judgment rate from 8.5 to 9.5 years, followed by an increase thereafter.

This study has several limitations. The small number of cases in this study may have led to an underestimation of the determination rate. Therefore, future studies are needed to confirm the results with a larger number of cases. In addition, this study included Japanese participants, and it is known that the color of the fundus varies among different races [50]. Therefore, the parameters with large sex differences may also differ by race, and the results of this study cannot be applied directly to other races.

In conclusion, we found that sex differences in the fundus gradually increased around the age of 10 years, the age at which children start developing secondary sexual characteristics, and the rate of accurate sex determination based on fundus parameters increased with increasing age. Relatively large sex differences in fundus photographs were found with regard to the color of the fundus and angles of the retinal arteries. Research on sex differences in fundus photographs has just begun and should be expanded in the future to investigate other sex differences and their relationship to the fundus parameters.

## Supporting information

S1 Table Sex differences in the ocular fundus parameters used for the analyses. (XLSX)

S2 Table The raw data in this study. (XLSX)
